# Chemsex-associated drug use amongst men and gender-diverse people having sex with men in the UK: Findings from an online community survey, 2024

**DOI:** 10.1177/09564624261448075

**Published:** 2026-04-28

**Authors:** George Baldry, Giulia Habib Meriggi, Dolores Mullen, Helen Corkin, Amelia Andrews, Catherine M Lowndes, David Reid, Catherine H Mercer, John Saunders, Hamish Mohammed, Dana Ogaz

**Affiliations:** 1 Blood Safety, Hepatitis, STI & HIV Division, 371011 UK Health Security Agency, London, UK; 2 Institute for Global Health, University College London, London, UK; 3 The National Institute for Health and Care Research Health Protection Research Unit in Blood Borne and Sexually Transmitted Infections At University College London in Partnership with the UK Health Security Agency, London, UK

**Keywords:** chemsex, GBMSM, drug use, sexual health, United Kingdom

## Abstract

**Objectives:**

Chemsex is the use of select psychoactive drugs to enhance sexual experiences and has been described among gay, bisexual, and other men who have sex with men (GBMSM). We aimed to characterise sexual risk, wellbeing and health-seeking behaviours among GBMSM and gender-diverse people reporting chemsex-associated drug use.

**Methods:**

We analysed data from ‘Reducing inequalities in Sexual Health’ (RiiSH), an online community survey of 2758 UK-resident men and gender-diverse people having sex with men undertaken in November-December 2024. We compared those reporting chemsex-associated drug use with those who did not, assessing sociodemographic characteristics, well-being, sexual risk behaviours and sexual health service (SHS) engagement.

**Results:**

Overall, 8% (218/2758) reported chemsex-associated drug use in the last year. A higher proportion of participants reporting chemsex-associated drug use in the last year also reported a composite measure of sexual risk based on self-reported behaviours in the previous 3-4 months (e.g. prior bacterial STI diagnosis, ≥5 male condomless anal sex partners) compared to those who did not (85% vs 61%, *p* < 0.001). They also more frequently reported attending a SHS in the last year (81% vs 57%, *p* < 0.001). Those reporting chemsex-associated drug use also more frequently reported a long-term limiting mental health condition (36% vs 24%, *p* < 0.001) and poorer personal wellbeing (e.g. reporting low life satisfaction 36% vs 20%, *p* < 0.001).

**Conclusion:**

While a minority of participants in this national, community-based sample reported chemsex-associated drug use, this group had higher sexual risk and poorer indicators of wellbeing. Many participants also attended SHSs, reinforcing the key supporting role of SHSs for referral pathways to harm reduction support for those experiencing problematic drug use.

## Introduction

Chemsex refers to the use of drugs before or during planned sexual activity to sustain, enhance, disinhibit or facilitate the experience. Chemsex generally relates to the use of specific substances, and while there is no universally used definition, it commonly involves use of gamma-hydroxybutyrate/gamma-butyrolactone (GHB/GBL), crystal methamphetamine (‘crystal meth’), or mephedrone, and is reported by some gay, bisexual, and other men who have sex with men (GBMSM).^1,2^ While not all drug use associated with chemsex is inherently problematic,^1,3^ chemsex is associated with increased sexual risk-taking, such as condomless anal sex (CAS) and multiple concurrent sexual partners,^4^ which increase the risk of sexually transmitted infection (STI) and blood-borne virus (BBV) transmission.^5^ Some studies also found lower levels of HIV testing among people engaging in chemsex.^4^ Beyond sexual health risks, use of these drugs, particularly when injected intravenously (‘slamming’),^6^ has been associated with wider harms, including substance dependence, sexual assault or abuse, and lifestyle harms, such as loss of employment.^6^ Similar patterns of chemsex-associated drug use and associated health impacts in GBMSM have also been described across Europe, North America, Asia and Australia.^6,7,8^ However, there is a lack of recent epidemiological data related to chemsex in the UK. A more up to date understanding of the picture of chemsex, as well as access to and use of services to support those that require help, is important.

Using data from a large, online cross-sectional survey conducted in 2024, we aim to estimate the prevalence of chemsex-associated^6^ drug use and describe the demographic, behavioural and wellbeing characteristics of participants using these drugs among a community sample of men and gender-diverse people having sex with men in the UK.

## Methods

The ‘Reducing inequalities in Sexual Health’ (RiiSH) survey is a series of annual online cross-sectional surveys, assessing the sexual health and well-being of a UK community sample of men and gender-diverse people who have sex with men. For RiiSH-2024, participants were recruited through advertisements on social networking sites and geospatial dating platforms from 18^th^ November-11^th^ December 2024. Participants eligible to take part included self-identifying men (cisgender or transgender), transgender women or gender-diverse people who were assigned male at birth (AMAB), aged ≥16 years, residing in the UK and reporting sex with a man or non-binary AMAB person in the last year.

We examined chemsex-associated drug use in all RiiSH-2024 participants. Participants were asked if they had ever taken crystal meth, mephedrone or GHB/GBL. These three drugs were chosen using a combination of existing literature and data on the patterns of chemsex drug use in the UK. If they reported ever using these drugs, they were asked for the recency of last use (over 1 year ago, in the last year, or since August 2024 [in the last 3-4 months]). Those who reported use in the previous 3-4 months were asked what proportion of their sex involved chemsex-associated drug use on a scale from none to all. All participants were asked separately if they had ever injected drugs, excluding prescribed medicines or anabolic steroids.

We present the proportion of participants who reported chemsex-associated drug use, ever and in the last year. Differences in the sociodemographic characteristics (age-group, sexual orientation, ethnicity, area of residence, financial comfort, country of birth, education, employment), clinical history (HIV status, ever use of online postal self-sampling kits for STI testing, SHS attendance in last year), sexual risk behaviour (see below), and personal-wellbeing (life satisfaction, long-term limiting physical or mental health conditions) among those reporting and not reporting chemsex-associated drug use were examined using Pearson’s chi-squared test as all data were categorical. Given the descriptive aim of the study and a priori concerns regarding small subgroup sizes among those reporting chemsex-associated drug use, regression modelling was considered outside the scope of the analysis.

A composite measure of sexual risk was used in analyses, defined by reporting any of the following within the last 3-4 months: a bacterial STI diagnosis (*Chlamydia trachomatis, Neisseria gonorrhoeae and Treponema pallidum* [syphilis]), ≥5 male condomless anal sex (CAS) partners, and meeting partners through sex-on-premises venues, public sex environments (PSE i.e. cruising environments), or at private sex parties, and/or HIV pre-exposure prophylaxis (PrEP) use in the last year. We also report sexual risk behaviours included in the composite measure separately.

To assess wellbeing, we used UK’s Office for National Statistics’ (ONS) personal well-being indicators, including a dichotomised measure of low life-satisfaction based on ONS harmonisation standards (i.e. low vs medium, high, very high).^9^ For participants reporting a long-term physical or mental health conditions, we derived a binary variable indicating limitations. Participants who responded ‘Yes, a little’, ‘Yes, a lot’ (vs ‘Not at all’) to the question, “Does your condition or illness reduce your ability to carry out day-to-day activities?” were classified as having a limiting condition. Those not reporting a long-term health condition or who did not indicate limitations were classified as having no limitation. We created a binary variable to assess the frequency of sex under the influence of alcohol in the last year (over half of sex vs less than half).

## Results

There were 2758 participants included in analyses, with a median age of 45 (IQR: 36-55). Most were cisgender men (95%), gay (80%), of white ethnicity (88%), and lived in England (87%). The majority were employed (78%), and degree-educated (59%) (Table 1).

**Table 1.** Sociodemographic characteristics, sexual risk behaviours and service use in participants reporting and not reporting chemsex-associated drug use in the last year.

	All participants	No chemsex-associated drug use in last year	Chemsex-associated drug use in last year	*p*-value
Number of respondents	2758 (100.0%)	2540 (92.1%)	218 (7.9%)	
Age (years) at survey completion (median IQR)	45 (36-55)	45 (36-55)	45 (37-55)	
**Sociodemographic characteristics**				
Age-group (years)				0.713
16–29	337 (12.2%)	314 (12.4%)	23 (10.6%)	
30–44	1010 (36.6%)	927 (36.5%)	83 (38.1%)	
45 and over	1411 (51.2%)	1299 (51.1%)	112 (51.4%)	
Sexual orientation				0.006
Gay/homosexual	2207 (80.0%)	2017 (79.4%)	190 (87.2%)	
Bisexual, straight, or other^a^	551 (20.0%)	523 (20.6%)	28 (12.8%)	
Ethnic group				0.063
White	2435 (88.3%)	2251 (88.6%)	184 (84.4%)	
Another ethnic group	323 (11.7%)	289 (11.4%)	34 (15.6%)	
Area of residence				<0.001
Outside London/unknown (England)	1647 (59.9%)	1556 (61.5%)	91 (41.9%)	
Lives in London	748 (27.2%)	646 (25.5%)	102 (47.0%)	
Outside England	354 (12.9%)	330 (13.0%)	24 (11.1%)	
Comfortable financial situation				<0.001
No	1512 (54.8%)	1363 (53.7%)	149 (68.3%)	
Yes (top two quartiles)^b^	1246 (45.2%)	1177 (46.3%)	69 (31.7%)	
UK Born				<0.001
No	579 (21.0%)	513 (20.2%)	66 (30.3%)	
Yes	2179 (79.0%)	2027 (79.8%)	152 (69.7%)	
Degree-level education				0.294
No	1130 (41.0%)	1048 (41.3%)	82 (37.6%)	
Yes	1628 (59.0%)	1492 (58.7%)	136 (62.4%)	
Employed				0.004
No	619 (22.4%)	553 (21.8%)	66 (30.3%)	
Yes	2139 (77.6%)	1987 (78.2%)	152 (69.7%)	
**Clinical history**				
HIV Status				<0.001
HIV negative/unknown	2458 (89.1%)	2298 (90.5%)	160 (73.4%)	
PLWHIV (tested positive)	300 (10.9%)	242 (9.5%)	58 (26.6%)	
Ever used online self-sampling kits for STI testing				0.192
No	1720 (62.4%)	1593 (62.7%)	127 (58.3%)	
Yes	1038 (37.6%)	947 (37.3%)	91 (41.7%)	
Visited SHS in last year				<0.001
No	1134 (41.1%)	1093 (43.0%)	41 (18.8%)	
Yes	1624 (58.9%)	1447 (57.0%)	177 (81.2%)	
**Behavioural characteristics**				
Composite measure of sexual risk ^c^				<0.001
No	1035 (37.5%)	1002 (39.4%)	33 (15.1%)	
Yes	1723 (62.5%)	1538 (60.6%)	185 (84.9%)	
PrEP use in the last year^d^				<0.001
No PrEP use in last year	1215 (49.4%)	1179 (51.3%)	36 (22.5%)	
PrEP use in last year	1243 (50.6%)	1119 (48.7%)	124 (77.5%)	
Number of male condomless anal sex partners (CAS) in last 3-4 months				<0.001
<5 CAS partners	2086 (75.6%)	1981 (78.0%)	105 (48.2%)	
≥5 CAS partners	672 (24.4%)	559 (22.0%)	113 (51.8%)	
Bacterial STI diagnosis in last 3-4 months^e^				<0.001
No	2487 (90.2%)	2329 (91.7%)	158 (72.5%)	
Yes	271 (9.8%)	211 (8.3%)	60 (27.5%)	
Met partners at sex-on-premises venues, cruising sites or sex parties in last 3-4 months				<0.001
No	1847 (67.0%)	1746 (68.7%)	101 (46.3%)	
Yes	911 (33.0%)	794 (31.3%)	117 (53.7%)	
Ever injected any drug other than prescribed medicines or anabolic steroids				<0.001
No	2648 (96.0%)	2500 (98.4%)	148 (67.9%)	
Yes	110 (4.0%)	40 (1.6%)	70 (32.1%)	
Frequency of sex under the influence of alcohol in the last year				0.004
Less than half or none	2443 (88.6%)	2263 (89.1%)	180 (82.6%)	
At least half	315 (11.4%)	277 (10.9%)	38 (17.4%)	
Wellbeing				
Long-term limiting mental health condition				<0.001
No	2078 (75.3%)	1939 (76.3%) (79.2%)	139 (63.8%)	
Yes	680 (24.7%)	601 (23.7%)	79 (36.2%)	
Long-term limiting physical health condition				0.167
No	2176 (78.9%)	2012 (79.2%)	164 (75.2%)	
Yes	582 (21.1%)	528 (20.8%)	54 (24.8%)	
Low life satisfaction				<0.001
No (medium, high, very high satisfaction)	2159 (78.4%)	2019 (79.6%)	140 (64.2%)	
Yes	594 (21.6%)	516 (20.4%)	78 (35.8%)	

^a^ Includes those identifying as bisexual, straight/heterosexual, or another way.

^b^ Top two quartiles (“I am comfortable”/“I am very comfortable” from the question, “How would you best describe your current financial situation”.

^c^ Composite measure of sexual risk defined by reporting any of the following within the last 3-4 months: a bacterial STI diagnosis (chlamydia/gonorrhoea/syphilis), having ≥5 condomless anal (CAS) sex partners, and meeting partners through sex-on-premises venues, public sex environments (i.e. cruising environments), or at private sex parties, and/or HIV pre-exposure prophylaxis (PrEP) use in the last year.

^d^ Reported in those not known to be living with HIV.

^e^ Diagnosis of chlamydia, gonorrhoea, and/or syphilis.

PrEP = HIV pre-exposure prophylaxis. PLWHIV = people living with HIV. STI = sexually transmitted infection. SHS = sexual health service. CAS = condomless anal sex. RiiSH = 'Reducing inequalities in Sexual Health’. Chemsex-associated drug use = self-reported use of crystal methamphetamine, mephedrone or gamma-hydroxybutyrate/gamma-butyrolactone.

Overall, 15% (408/2758) reported chemsex-associated drug use ever in their lifetime, and 8% (218/2758) reported use in the last year (or 53% [218/408] of those reporting use ever). Among 138 participants reporting chemsex-associated drug use in the last 3-4 months, 90% (123/136, where specified by the participant) indicated that they used these drugs during some of their sexual encounters over the same period; among whom, 65% (80/123) reported use in at least half of the sexual encounters they had. Among all participants, 4% (110/2758) reported ever injecting any non-prescribed drugs, with 41% of these participants (45/110) reporting that they had injected drugs in the in the last 3–4 months.

The median age of those reporting chemsex-associated drug use in the last year (n = 218) was 45 years (interquartile range [IQR]: 37-54), similar to those not reporting use (45 [IQR:36-55]) (Table 1). A higher proportion of those reporting chemsex-associated drug use identified as gay (87% vs 80% in those reporting no use, *p* = 0.006), resided in London (47% vs 26%, *p* < 0.001) and were living with HIV (27% vs 10%, *p* < 0.001). People using chemsex-associated drugs more commonly reported having a limiting long-term mental health condition (36% vs 24%, *p* < 0.001) or low life satisfaction (36% vs 20%, *p* < 0.001). A smaller proportion also reported being financially comfortable compared to those who had not used these drugs in the last year (32% vs 46%, *p* < 0.001).

**Figure 1.**
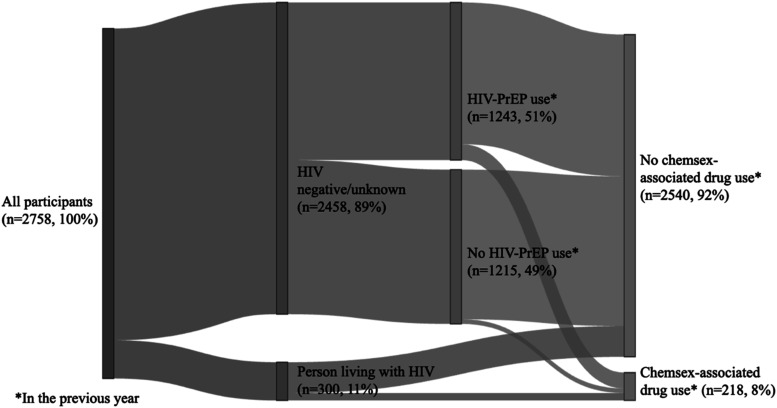
The proportion of people engaging in chemsex-associated drug use in the previous year, stratified by HIV status and HIV-PrEP use in the previous year.

These participants also more frequently reported our composite measure of sexual risk (85% vs 61%, p < 0.001) and attending a SHS in the last year (81% vs 57%, *p* < 0.001). Chemsex-associated drug use was also more frequently reported in those reporting HIV-PrEP use in the last year (78% vs 49%, *p* < 0.001, in those not known to be living with HIV), shown in Figure 1. Reporting that at least half of their sexual encounters in the last year was under the influence of alcohol was also more common in this group (17% vs 11%, *p* = 0.004).

## Discussion

While fewer than 1 in 10 men and gender-diverse people having sex with men reported chemsex-associated drug use in the previous year, this group had high levels of sexual risk and poorer wellbeing, including limiting long-term mental health conditions. Findings affirm that chemsex-associated drug use remains an important public health issue given a range of intersectional vulnerabilities and need for tailored harm reduction and holistic support.

The prevalence of chemsex-associated drug use (15% ever, 8% in the last year) and co-occurring characteristics appear consistent with reporting prior to the widespread disruptions caused by COVID-19 in similar community samples.^4,10^ Chemsex-associated drug use was more common among participants who identified as gay, those residing in London, and among those living with HIV. Nearly half of participants reporting use lived in London, aligning with prior London-focused studies^11,12^ and providing ongoing evidence of higher prevalence in urban settings.^13,14^ Patterns of use may reflect the influence of higher numbers of GBMSM residing in larger UK cities,^15^ and established sexual networks that use chemsex-associated drugs. One previous study of chemsex among GBMSM living with HIV, found that associations between living in London and participating in chemsex were not significant after adjusting for high-risk sexual behaviours. This may indicate that chemsex represents one aspect of higher sexual risk-taking that may be generally more common in London, rather than chemsex alone being associated with urban centres.^16^ Although our survey collected information on participants’ area of residence, we were unable to distinguish rural and urban settings. Consequently, we were unable to understand how area of residence or location of sexual activity was associated with chemsex-associated drug use.

These findings demonstrate a range of potential negative outcomes that may accompany chemsex-associated drug use, alongside patterns of risk-taking behaviours and potential wellbeing needs. Participants who reported chemsex-associated drug use also reported higher levels of sexual risk behaviours, including condomless anal sex partnerships, and meeting partners at sex-on-premise venues.^3^ This group also reported lower wellbeing indicators, such as low life satisfaction, and having a limiting long-term mental health condition. A higher frequency of sex under the influence of alcohol was also more common in those reporting chemsex-associated drug use. Other potential wellbeing consequences such as negative impacts on work, and relationships with friends and family were outside the scope of this survey, but have been previously been previously associated with GBMSM living with HIV who were engaging in chemsex.^17^ These factors may act either as drivers or consequences of concurrent sex and drug use.^3^ Irrespective of directionality, they could indicate persistent intersectional vulnerabilities, which are important factors to consider when designing and implementing effective interventions for those reporting chemsex-associated drug use.

Those reporting chemsex-associated drug use had greater engagement with SHSs in the last year and higher levels of PrEP use. Similar self-mitigation of risk is consistently reported across literature,^3,4,18^ including higher levels of STI testing, PrEP use, and intentional structuring of drug use (e.g. dosage control and avoidance of injecting practices). While these findings suggest that those using chemsex drugs are aware of their elevated risk and seek to mitigate it through SHS engagement, their service use may still be driven by their sexual health needs, not directly related to chemsex-associated drug use.

### Implications of findings

High engagement with SHSs among participants reporting chemsex-associated drug use suggests the importance of these settings as key points of contact for assessing and addressing chemsex-related care needs. Qualitative work in England indicates that SHSs are often preferred by service users over traditional drug misuse services, which may be perceived as more stigmatising.^19^ Greater shifts towards digital sexual health service delivery may further complicate appropriate support or referral for chemsex-related needs, and should consider appropriate triage.^20^

Although chemsex-associated drug use was more commonly reported in London, our findings indicate chemsex-associated drug use across the UK. It is therefore essential to also ensure adequate healthcare and social infrastructure to meet the needs of people using chemsex-associated drugs throughout the UK which minimises individual and policy-level barriers. Holistic, culturally-competent care, including mental health and psychosocial interventions, that address stigma and shame associated with seeking help for problematic chemsex-associated drug use could play a role in meeting the needs of this population. Support approaches could include harm reduction education e.g. safer injection practices and safer use of substances such as GHB, while fostering sex positivity that promotes intimacy, connection and pleasure.^18^

### Strengths and limitations

We used data from a convenience sample of people recruited using social media and dating applications, where participants are more likely to report sexual risk behaviours than the general GBMSM population.^21^ Our estimate of chemsex-associated drug use (ever) may be overestimated given the older age-distribution of participants. However, drawing from a community sample may enhance generalisability compared to studies relying solely on SHS recruitment. Given the small number of participants reporting chemsex-associated drug use, adjusted analyses to account for confounding were not performed. The RiiSH survey did not collect any identifiers such as name or address to reduce social desirability bias, and this may have facilitated reporting of reported chemsex-associated drug use and injection practices. Still, this study lacks diversity in key demographic areas (e.g. ethnicity, age, sexual and gender identity) limiting our ability to assess chemsex-associated drug use in more marginalised groups. This also resulted in a large proportion of the sample being London residents. This likely reflects the larger population of GBMSM residing in London,^22^ particularly those with a high degree of SHS engagement. While our findings on the proportion of GBMSM reporting chemsex-associated drug use are consistent with previous studies,^4,10^ the selection bias in our sample affects generalisability and limits our ability to identify any demographic and behavioural differences inside versus outside London.

As these data were self-reported, there may also be differences in perception between participants if questions were subjective or related to personal experience (e.g. “Does your condition or illness reduce your ability to carry out day-to-day activities?”). This may have resulted in people reporting different responses for questions about long-term physical or mental health conditions despite similar levels of experienced limitations. However, our use of binary variables (grouping ‘Yes, a little’ and ‘Yes, a lot’) may have reduced any misclassification bias in responses to these questions.

## Conclusion

While the proportion of men and gender-diverse people engaging in chemsex-associated drug use was relatively low, the more frequently reported levels of sexual risk behaviours, which can increase STI and BBV transmission risk, highlights the need for targeted interventions. Patterns of use and reported risks appear largely consistent with studies conducted before the COVID-19 pandemic. Effective harm reduction must tackle a wide spectrum of needs, including general and sexual wellbeing, and mental health. These efforts must be implemented within diverse healthcare settings and not be lost in digital delivery.

Given the intricate sociocultural landscape in which chemsex occurs, interventions to reduce the public health impact of associated harms should be built collaboratively, incorporating meaningful community coproduction. This can empower people to enhance sexual wellbeing, reduce harm, and access appropriate care, while also strengthening the capacity of SHSs and voluntary sector organisations to respond to needs appropriately within the UK and internationally.

## Data Availability

The data that support the findings of this study are not publicly available to protect participant privacy. However, some aggregate data are available upon reasonable request from the UK Health Security Agency (UKHSA). Requests can be directed to DataAccess@ukhsa.gov.uk.
